# Polymer for Internal Hydrophobization of Cement-Based Materials: Design, Synthesis, and Properties

**DOI:** 10.3390/polym13183069

**Published:** 2021-09-11

**Authors:** Xiao Liu, Xiaofei Song, Ziming Wang, Chunlei Xia, Ting Li, Xiaoning Li, Qian Xu, Suping Cui, Shanshan Qian

**Affiliations:** 1 Faculty of Materials and Manufacturing, Key Laboratory of Advanced Functional Materials, Ministry of Education, Beijing University of Technology, Beijing 100124, China; songxf@emails.bjut.edu.cn (X.S.); wangziming@bjut.edu.cn (Z.W.); xuqian@ctc.ac.cn (Q.X.); cuisuping@bjut.edu.cn (S.C.); aritqss@163.com (S.Q.); 2 Beijing Municipal Engineering Research Institute, Beijing 100037, China; Xiachunlei2005@163.com; 3 CNBM Zhongyan Technology Co., Ltd., Beijing 100024, China; liting1867@126.com (T.L.); lixiaon.good@163.com (X.L.); 4 China Building Material Test & Certification Group Co., Ltd., China Building Materials Academy, Beijing 100024, China; 5 Faculty of Materials and Manufacturing, National Engineering Laboratory for Industrial Big-Data Application Technology, Beijing University of Technology, Beijing 100124, China; 6 Jiangsu ARIT New Materials Co., Ltd., Nanjing 211505, China

**Keywords:** hydrophilicity–hydrophobicity conversion, internal hydrophobicity, molecular design, cement-based material, hydration

## Abstract

A series of novel comb-like poly(butyl acrylate)-g-poly(dimethylaminoethyl methacrylate) (PBA-g-PDMAEMA) with different side chain lengths were designed and successfully synthesized by the “first main chain then side chain” method. Infrared Spectroscopy (IR), ^1^H Nuclear Magnetic Resonance (^1^H NMR), and gel permeation chromatography (GPC) were used for structural confirmation and molecular weight characterization. This polymer exhibited responsive behavior from hydrophilicity to hydrophobicity under the alkaline environment of cement-based materials, with the contact angle of 105.6°, a decreased evaporation rate, and a hydrophile–lipophile balance (HLB) value. A significant internal hydrophobic effect on cement mortar was shown in the water absorption rate, which decreased by 75.2%, and a dry shrinkage-reducing rate of more than 30%. Furthermore, this polymer can effectively slow the exothermic rate, reduce the heat release, and delay the exothermic peak of cement hydration. It was interesting that these properties showed a direct correlation with the side chain length of the comb polymer. The aims of this study are to provide a new avenue to synthesize polymers with the spontaneous hydrophilicity–hydrophobicity transition in the cement system, achieving excellent internal hydrophobicity of cement-based materials, and to offer a promising alternative to resist external erosion for improving the durability and service life of cement-based materials.

## 1. Introduction

With the development of construction engineering technology, concrete materials play a very important role in the construction of modern projects, and have become more indispensable [1] due to the advantages of a high strength, easy casting, low cost, etc. Based on the requirements of major key projects, concrete science and technology is developing towards the direction of high quality, high yield, and special performance [2]; however, there are still some defects in concrete itself. The shrinkage of concrete leads to internal cracking [3,4], which will reduce the strength, make it easier for external harmful substances to enter the interior, and accelerate the deterioration of the durability of concrete, thus affecting the quality of the project and the service life of the building [5,6]. Therefore, it is necessary to restrain the shrinkage cracking of concrete and improve the durability of concrete.

Water is the key factor of reducing the durability of concrete structures [7]. Concrete with microcracks and pores provides a way for the transport of corrosive substances in concrete, such as Cl^−^ [8], CO_2_ [9,10], sulfate [11], etc. With the infiltration of water into the concrete, the hydrostatic pressure inside the concrete increases, and moreover, the alkali aggregate reaction, steel corrosion, water icing, etc., will destroy the volume stability and durability of the concrete. To reduce the shrinkage and cracking of concrete and extend its service life, water-tight and self-compacting concrete [12] can be used to reduce the entry of water and other corrosive substances. At present, the more effective technical approaches to protect the concrete structures mainly include: a coating surface protection layer [13,14] to avoid or limit water into the concrete interior, or adding an emulsion admixture [15] to reduce internal capillary pressure.

It is generally believed that hydrophobic impregnation can establish an effective physical barrier for concrete, thereby retarding the corrosion and reducing the internal corrosion rate [16]. This has also been well demonstrated to be effective in greatly reducing internal humidity, capillary water absorption, and the invasion of water-soluble salts, such as Cl^−^. This even allows water vapor to enter and exit, making the concrete “breathe” freely [17]. Furthermore, hydrophobic treatment increases the water repellency of the surfaces of cement-based materials without filling the pores, which not only counteracts but also reverses the capillary suction, thus preventing the penetration of liquid water, even when the water is pressurized [18].

Organic film coating is also an effective method to improve the surface hydrophobicity and corrosion resistance of concrete [19,20]. This coating, composed of polymer compounds, forms a protective barrier between the concrete and the external environment, thus effectively preventing or delaying the deterioration of concrete. The polymer compounds such as acrylic acid [21], chlorinated rubber, epoxy resin, and polyurethane [22] can form a continuous organic film coating on the surfaces of concrete to prevent the invasion of harmful substances, which is helpful to significantly improve the durability of concrete. However, the protective effect of coatings on concrete is usually reduced due to a combination of sunlight, heat, water, salt, or alkaline pore solutions [20,23]. Once the protective film coating is damaged, aged, or ineffective, this method will lose its significance. In addition, this surface hydrophobic treatment is different from an internal hydrophobic treatment, which can fundamentally prevent the erosion of harmful substances, even when the surface film coating fails [24]. As for internal hydrophobic treatments [25,26], if an emulsion admixture is used, there are still many problems, such as a complex system, uncontrollable particle size, easy phase separation, and difficult removal of the emulsifier and impurity, etc. Therefore, how to design and develop new polymers to achieve real internal hydrophobic modification has become the key technology to improve the durability and service life of cement-based materials. However, there have been few studies reported on this.

In this study, the novel comb-shaped polymer was designed and synthesized through the “formation of main chain” from the self-polymerization of carboxylate monomers and the “graft of side chain” from the graft copolymerization of responsive monomers onto main chains. A series of such polymers with different side chain lengths were synthesized by adjusting the molar ratio of the main chain monomer to the side chain monomer. The molecular structures of the synthesized polymers were confirmed by Infrared spectroscopy (IR) and ^1^H Nuclear Magnetic Resonance (^1^H NMR), and the molecular properties were determined by gel permeation chromatography (GPC). Then, the hydrophobicity behavior, contact angle, evaporation rate, and hydrophile–lipophile balance (HLB) value of the polymer solution were investigated. In order to verify the internal hydrophobic application effect of these polymers on cement-based materials, the water absorption, shrinkage, and strength of cement mortars containing polymers with different side chain lengths were evaluated. Furthermore, the influence on cement hydration and hydration products was analyzed by the measurements of hydration heat and X-ray diffraction (XRD). This study provides not only a new guidance for developing novel polymers, but also a new technical direction for improving the durability and service life of cement-based materials.

## 2. Materials and Methods

### 2.1. Materials

Butyl acrylate (BA, ≥99% purity) was purchased from Shanghai McLean Biochemical Technology Co., Ltd. (Shanghai, China). Moreover, 2-(dimethylamino) ethyl methacrylate (DMAEMA), 2,2′-azobis(2,4-dimethyl) valeronitrile (ABVN), and 1,4-xylene (PX) (all ≥99% purity) were purchased from Shanghai Aladdin Biochemical Technology Co., Ltd. (Shanghai, China). Benzoyl peroxide (BPO, ≥98% purity) was purchased from Tianjin Fuchen Chemical Reagent Factory (Tianjin, China). The reference cement P.I.42.5 was provided by China Building Materials Research Institute (Beijing, China), and its chemical composition and mineral composition are shown in Table 1. China ISO standard sand was mainly provided by Xiamen ISO standard sand Co., Ltd. (Xiamen, China). The water reducing agent used in this experiment is a commercially available polycarboxylate superplasticizer (PCE).

### 2.2. Molecular Design

In this study, a comb-shaped polymer was designed, whose main chain could be hydrolyzed in a cement solution environment system to produce anchoring groups that adsorbed on the surfaces of cement particles. Additionally, its side chains could produce the conversion from hydrophilicity to hydrophobicity in the cement solution environment. Hence, BA was selected as the monomer of the main chain due to its water solubility and its ester group. The ester group is the electron-donating group that is easy to produce active grafting points and high grafting efficiency, and can be hydrolyzed to produce a carboxyl group in the alkaline environment of the cement solution for a certain anchoring effect on the cement particles [27,28]. Also, DMAEMA was selected as the monomer of the side chains for its water solubility and its responsive behavior to hydrophobicity by tertiary amine groups, which show hydrophilicity in both acidic and neutral environments due to protonation, while showing hydrophobicity in an alkaline environment of cement due to deprotonation [29,30]. Besides, this monomer is not easy to hydrolysis in an alkaline environment.

Herein, BPO was selected as the initiator for the self-polymerization process of the main chain, since it is suitable for polymerization in the oil-soluble system at a high temperature, such as above 100 °C [31]. As for ABVN, it was selected as the initiator for the graft copolymerization process of the side chains due to its higher activity at medium and low temperatures [32]. The mixture of PX and 200# paint solvent was selected as the solvent to reduce the viscosity of the system and to control the molecular weight of the polymer by chain transfer.

The whole reaction process was divided into two steps: (1) the self-polymerization of the main chain, wherein BA was self-polymerized under the initiator of BPO to form poly butyl acrylate (PBA); and (2) the graft copolymerization of the side chain, wherein DMAEMA were graft-polymerized onto the synthesized PBA under the ABVN initiator to generate PBA-g-PDMAEMA. The grafting points of the side chains were on the tertiary hydrogen atoms of the main chain.

### 2.3. Synthesis

#### 2.3.1. Synthesis of PBA

**Figure 1:**
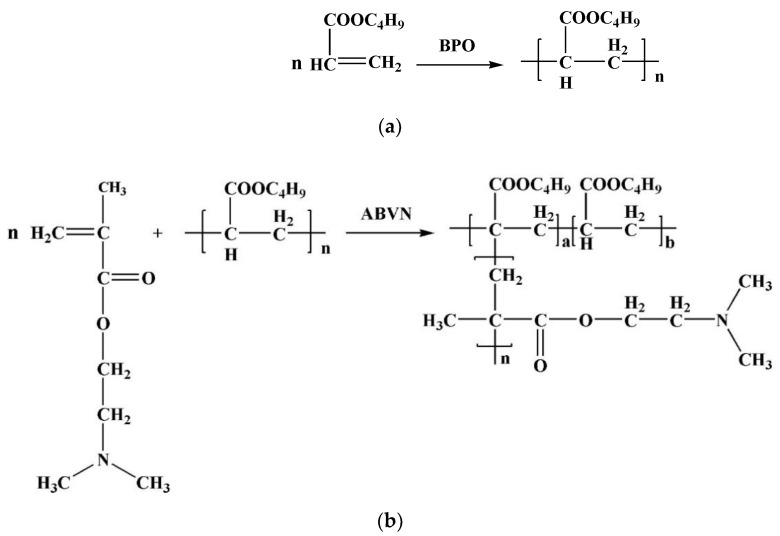
Chemical equations: (**a**) self-polymerization of the main chain; (**b**) graft copolymerization of the side chains.

PX and 200# paint solvent (m_PX_:m_paint solvent_ = 1:1) were added to a four-neck round-bottom flask placed in a constant temperature bath at 100 °C with stirring, and then BA and BPO (m_BPO_:m_BA_:m_solvent_ = 1:50:125) were added dropwise to the mixture for 1.5 h, followed by stirring at a constant temperature for 1 h. The products were washed with water three times to get the solution of PBA. The chemical equation is shown in Figure 1a.

#### 2.3.2. Synthesis of PBA-g-PDMAEMA

The mixed solution of DMAEMA and ABVN (n_ABVN_:n_DMAEMA_ = 1:50) was added dropwise to the solution of PBA for 6 h at 40 °C with stirring, followed by stirring at a constant temperature for 6 h. After the reaction, the solvent was removed by vacuum distillation to get the PBA-g-PDMAEMA. The chemical equation is shown in Figure 1b.

PBA-g-PDMAEMAs with different side chain lengths were obtained by adjusting the molar ratio of the DMAEMA monomer to the BA monomer, i.e., 1:1, 5:1, 15:1, 20:1, and 25:1, which were also identified as sample numbers.

### 2.4. Structural Characterizations

#### 2.4.1. IR Analysis

IR measurements of the samples were performed on a VERTEX 70 Fourier transform infrared spectrometer (Bruker Co., Billerica, MA, USA). Spectra were collected in the region of 4000–400 cm^−1^ region with 32 scans at a resolution of 4 cm^−1^ and at 25 °C.

#### 2.4.2. ^1^H NMR

The ^1^H NMR spectrum of the samples was recorded on an ASCEndTM400 (AVANCEHD III) NMR spectrometer (Bruker Co., Billerica, MA, USA) operating at a frequency of 400 MHz, and the chemical shift values were expressed in d values (ppm) relative to tetramethylsilane (TMS) as an internal standard. A sample for ^1^H NMR was prepared by dissolving in the solvent, i.e., deuterated water (D_2_O) as an internal reference.

#### 2.4.3. GPC

The weight average molecular weights (M_w_) of the samples were determined on a Waters PL-GPC50 chromatograph (Polymer Laboratories, England, UK). The mobile phase was 0.1 mol/L NaNO_3_ aqueous solution, with an injection volume of 100 μL and a flow rate of 1 mL/min.

### 2.5. Measurements

#### 2.5.1. Transmittance

A series of sample solutions with a concentration of 0.03 g/mL at different pH values (from 7.0 to 14.0) were poured into a cuvette and placed in the sample room of the photometer. Then the transmittance data of the experimental samples were recorded on a SolidSpec-3700 UV near-infrared spectrophotometer (Shimadzu Co., Kyoto, Japan) with a wavelength of 550 nm.

#### 2.5.2. Contact Angle

Moreover, 3 g of each of the five polymer solutions were taken, then dropped on different glass slides and put into an incubator. The wind speed was kept the same (1 m/s), and the temperature was set to 30 °C until the polymer solutions were dried. The contact angle of the polymer was determined on a JC2000 static contact angle instrument (Beijing Zhongyi Kexin Technology Co. Ltd., Beijing, China) with a 2 mL syringe used to control the same amount of drops each time. The maximum shape of the droplet was selected, and the frozen image was controlled within 10s after the droplet touched the surface of the glass. The contact angle between the droplet and the glass surface was measured by the angle measurement method, and the average value of multiple measurements was taken to reduce the test error.

#### 2.5.3. Evaporation Rate

Five polymer samples were diluted to 10% aqueous solutions of 60 g (m_0_), stirred evenly and placed in a beaker with a diameter of 5 cm, and then put into an incubator. The wind speed was kept the same (1 m/s), and the temperature was set to 30 °C. The mass (m_t_) of the polymer sample was measured for 24 h and 48 h, respectively. The evaporation rate (μ) was calculated according to Equation (1):

μ = (m_0_ − m_t_)/(S × t) (1)

where m_0_ was the initial mass of the polymer samples solution (g), m_t_ was the mass of the polymer samples solution for t hours (g), S was the area of evaporation pan (cm^2^), and t was the time of the polymer samples solution in the incubator.

#### 2.5.4. HLB Value

The HLB values of the polymer solutions were calculated by the method of inorganic group contribution, where the groups in the polymer are divided into organic groups (generally hydrophobic groups) and inorganic groups (generally hydrophilic groups). According to this, the inorganic and organic contribution values in polymer molecules can be judged [33,34] to calculate the HLB value according to Equation (2):

HLB = [(∑Inorganic group value)/(∑Organic group value)] × 10(2)

#### 2.5.5. Water Absorption Rate

The water absorption test was carried out according to the Chinese standard JGJ/T 70-2009, the “Standard for test method of performance on building mortar” [35]. A set of 70.7 mm × 70.7 mm × 70.7 mm mortar specimens were formed, with the mass ratio of cement to standard sand of 1:3 (m_cement_ = 600 g, m_sand_ = 1800 g), and the PBA-g-PDMAEMA dosage of 1% by weight of cement (bwoc). The fluidity of the mortar was set as 130–140mm to determine W/C, which was a measurement method of the Chinese standard GB/T 2419-2005 [36]. The water absorption rate of mortar is the ratio of the increased mass after the mortar water absorption to the initial mass of dry mortar, as calculated according to the equation of the Chinese standard JGJ/T 70-2009 [35]. 

#### 2.5.6. Dry Shrinkage

The test of dry shrinkage of cement mortars were carried out according to the Chinese standard JC/T 603-2004, the “Standard test method for drying shrinkage of mortar” [37]. A set of 25 mm × 25 mm × 280 mm mortar specimens were formed, with the mass ratio of cement to standard sand of 1:2 (m_cement_ = 500 g, m_sand_ = 1000 g), the PBA-g-PDMAEMA dosage of 1% bwoc, and the PCE dosage of 0.2% bwoc. The fluidity of the mortar was set as 130–140 mm to determine W/C, which was a measurement method of the Chinese standard GB/T 2419-2005 [36]. The dry shrinkage rate was calculated according to the equation of the Chinese standard JC/T 603-2004 [37].

From the obtained data of the dry shrinkage rate, the shrinkage-reducing rate can be calculated according to Equation (3) [38]:

λ_i_ = (S_o_ − S_i_)/S_o_ (3)

where λ_i_ was the shrinkage-reducing rate of the polymer sample at the age of n days (%), S_0_ was the dry shrinkage rate of the cement mortar specimen without a polymer sample at the age of n days (%), and S_i_ was the dry shrinkage rate of the cement mortar specimen with a polymer sample at the age of n days (%).

#### 2.5.7. Mechanical Strength

The strength tests of the cement mortar were carried out according to the Chinese standard GB/T 17671-1999, the “Method of testing cements-determination of strength” [39], by a CDT305-2 compression flexure testing machine (MTS Industrial System Co. Ltd., Shenzhen, China), with the controllable preloading load, loading speed, and pressure holding time. A set of 40 mm × 40 mm × 160 mm mortar specimens were formed, with the mass ratio of cement to standard sand of 1:3 (m_cement_ = 600 g, m_sand_ = 1800 g), and the PBA-g-PDMAEMA dosage of 1% bwoc. The fluidity of the mortar was set as 130–140 mm to determine W/C, which was a measurement method of the Chinese standard GB/T 2419-2005 [36].

#### 2.5.8. Hydration Heat

The hydration heat and hydration exothermic rate of cement pastes with or without a polymer were measured by using a TAM AIR08 Thermostat (Thermometric, Järfälla, Sweden). The mass of the cement paste sample to be tested was 3 g with a W/C of 0.4, and the dosage of PBA-g-PDMAEMA was 1% bwoc.

#### 2.5.9. XRD

The hydrated phase developments of hardened pastes containing a polymer (W/C = 0.4, PBA-g-PDMAEMA = 1% bwoc) at 3 days and 7 days were determined by the XRD patterns recorded on a Shimadzu XRD-7000 diffractometer (Shimadzu Co., Kyoto, Japan) from 5° to 70° (2θ) at a scan rate of 4°/min using Cu Kα radiation. In this testing, after the curing ages of hydration for 3 days and 7 days, respectively, the cement blocks were placed in isopropyl alcohol for 24 h to stop hydration, and then put into an incubator for 24 h. The wind speed was kept the same (1 m/s), and the temperature was set to 40 °C. Finally, the sample blocks were ground into a powder and then sifted through 200 mesh.

## 3. Results and Discussion

### 3.1. Molecular Structure

#### 3.1.1. IR Analysis

**Figure 2:**
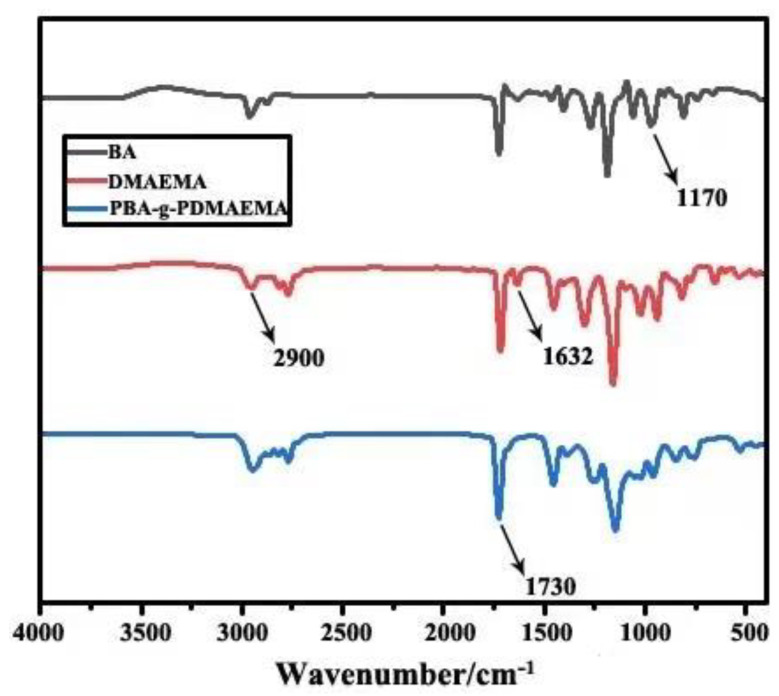
IR spectra of monomers and the copolymerization product.

Figure 2 shows the IR spectra of the main chain monomer, i.e., BA, the side chain monomer, i.e., DMAEMA, and the graft copolymerization product PBA-g-PDMAEMA, used for comparison. These spectra were analyzed according to the reported literature [40,41].

As can be seen from Figure 2, for BA, DMAEMA, and PBA-g-PDMAEMA, peaks all appeared at around 2900 cm^−1^, 1730 cm^−1^, and 1170 cm^−1^, which are stretching vibration peaks of –CH_2_–CH_2_–, –C=O, and –C–O–, respectively. By comparison, it was found that the peak for BA and DMAEMA both appeared at around 1632 cm^−1^, corresponding to the stretching vibration peak of –C=C–, while there was no peak at this position for PBA-g-PDMAEMA. This indicates that a polymerization reaction between BA and DMAEMA occurs, which is in line with the expected design. Detailed structural confirmation needs to be further determined by a ^1^H NMR study.

#### 3.1.2. ^1^H NMR Analysis

**Figure 3:**
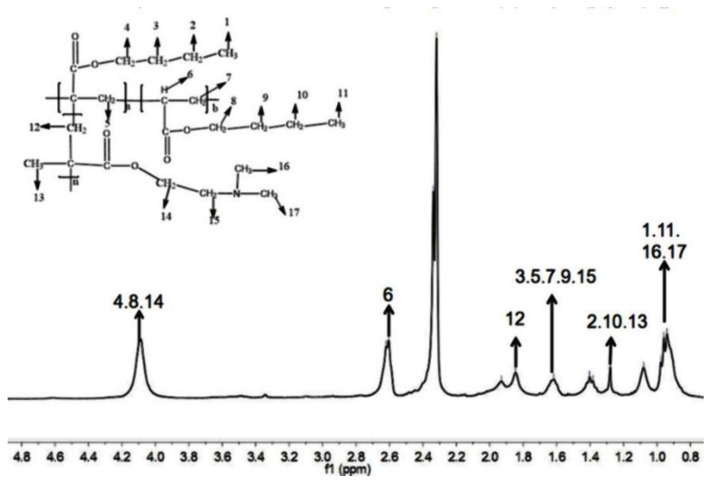
The ^1^H NMR spectrum of PBA-g-PDMAEMA synthesized by graft copolymerization.

Figure 3 shows the ^1^H NMR spectrum of the copolymerization product, which was analyzed by means of the reported chemistry book [42].

The structure diagram displayed in Figure 3 clearly indicates the characteristic H atoms in the molecular structure. It is well-known that the same groups in different molecular structures exhibit different peaks in the NMR spectrum. The key point from Figure 3 was that the chemical shift of the H atom of the –CH_2_– on the main chain of PDMAEMA was 1.5 ppm by calculation, but at this position, there was no peak shown in the NMR spectrum of PBA-g-PDMAEMA (Figure 3), signifying that the graft copolymerization reaction has occurred. In conclusion, a comb-shaped polymer PBA-g-PDMAEMA was successfully synthesized, which is in accordance with our expected design.

#### 3.1.3. Molecular Weight Analysis

The molecular weights of the main chain polymers and the grafted copolymerization samples are shown in Table 2.

As shown in Table 2, it can be found that: (1) the main chain polymer was successfully obtained by self-polymerization, and the number of structural units was about 68, which is in accordance with the experimental design; and (2) a series of graft copolymerization products with different side chain lengths were obtained by setting the molar ratios of the side chain monomer DMAEMA to the main chain monomer BA as 1:1, 5:1, 15:1, 20:1, and 25:1, respectively. With the increase of the molar ratio of DMAEMA to BA, the molecular weight and the side chain length of the graft copolymer increased, and reached the maximum when the molar ratio was 25:1. When the molar ratio increased to 30:1, the molecular weight decreased (data not shown here), which may be because an excessive amount of graft monomers leads to the entanglement of the side chains after reaching a certain length, and hinders the growth of the side chains on the other graft points of the main chain [43]. The above results further confirm that the comb-shaped structure can be obtained by grafting polymerization on tertiary carbon atoms, and such copolymers with different molecular weights can be obtained by adjusting the ratio of monomers.

### 3.2. Hydrophobicity Behavior

**Figure 4:**
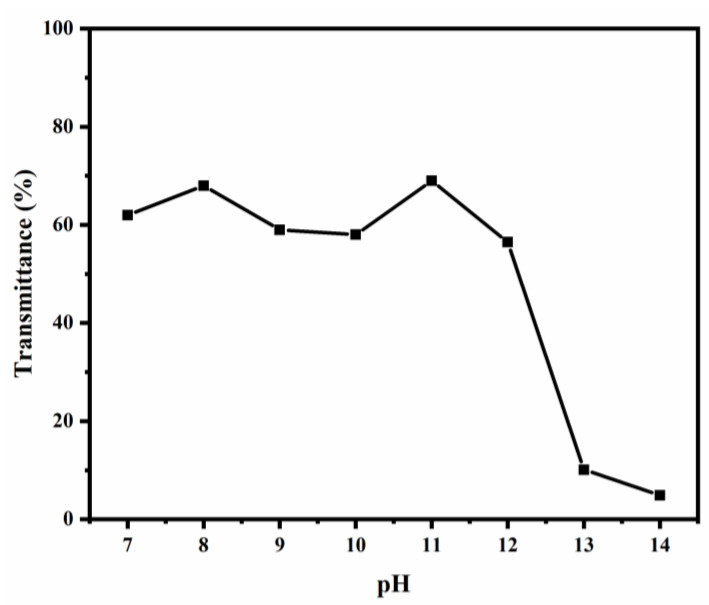
Transmittance of the PBA-g-PDMAEMA solution at different pH values.

To verify the response characteristics of the synthesized polymer in the solution, the transmittances of the PBA-g-PDMAEMA (sample 1:1) solution were measured under different pH values, as shown in Figure 4.

The PBA-g-PDMAEMA aqueous solution can perform responsive behavior in different chemical environments by means of the special characteristics of the tertiary amine groups of side chains. It exhibits hydrophilicity under acidic and neutral conditions due to protonation, presenting a high transmittance. On the other hand, a low transmittance as the form of hydrophobicity can be seen under an alkaline condition due to deprotonation.

As can be seen in Figure 4, the transmittance of the synthesized PBA-g-PDMAEMA sample was high when the pH value was 7–11, while the transmittance decreased sharply when the pH was 12–13, indicating good hydrophilicity in the pH range of 7–11, but good hydrophobicity in the pH range of 12–14. Therefore, it can be concluded that the conversion from hydrophilicity to hydrophobicity has been achieved, and interestingly, the pH range of the conversion is exactly equivalent to that of the alkaline environment of the cement slurry system with a pH range of about 12–13, which is in line with our design.

### 3.3. Hydrophobicity

**Figure 5:**
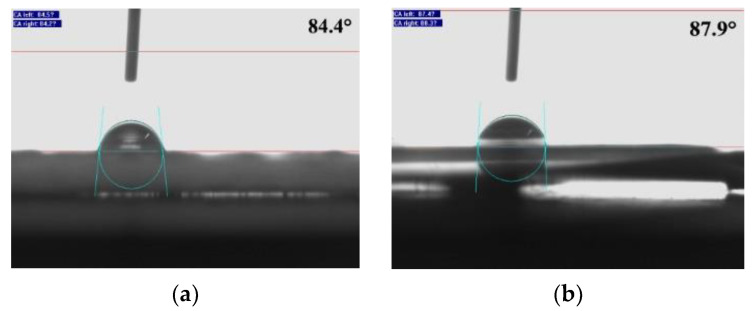

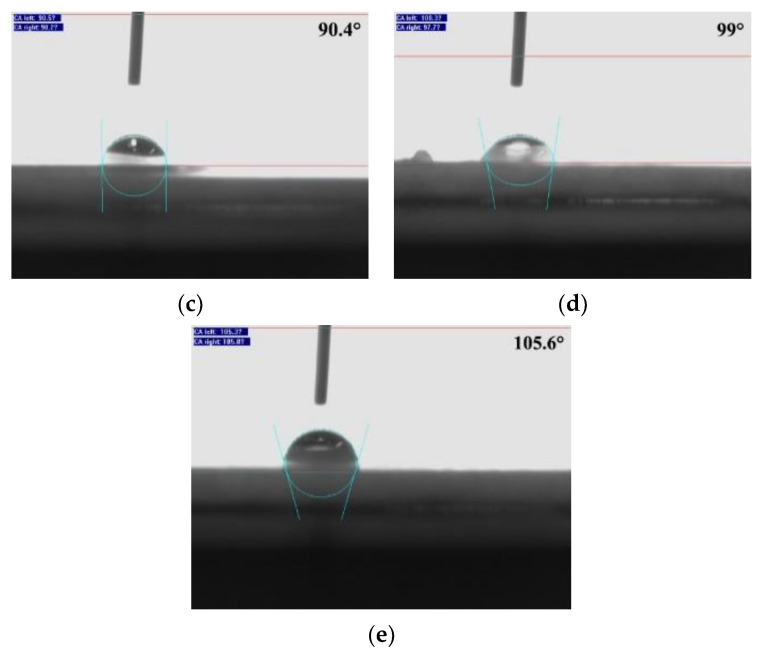
Contact angles of PBA-g-PDMAEMA sample at pH = 13: (**a**) 1:1, (**b**) 5:1, (**c**) 15:1, (**d**) 20:1, and (**e**) 25:1.

The contact angles of the synthesized PBA-g-PDMAEMA samples were measured by the static drop method using the drops of the aqueous solution (pH = 13). The captured images and the results of the contact angle (upper right corner of the image) are shown in Figure 5.

It is well known that a contact angle of less than 90° signifies hydrophilicity, while a contact angle of greater than 90° signifies hydrophobicity, and the smaller/larger the angle is, the stronger the hydrophilicity/hydrophobicity is [44]. From Figure 5a–e, the contact angles of the PBA-g-PDMAEMA samples were 84.4°, 87.9°, 90.4°, 99°, and 105.6°, which are much higher than that of the conventional hydrophilic state. This indicates that PBA-g-PDMAEMA can change its interface wetting state under an alkaline condition, showing significant hydrophobic characteristics. Interestingly, the contact angles of the samples exhibited a gradual increasing trend with the increase of the side chain length, showing that the contact angle is proportional to the side chain length of the comb polymer. The above results confirm the conclusion that the length of the functional side chain of the comb polymer PBA-g-PDMAEMA is an important factor affecting hydrophobicity. For the sample with the longest side chain, in Figure 5e, the contact angle exceeded 100°, indicating a significant hydrophobic effect and a strong conversion capability.

### 3.4. Solution Properties of Polymer

#### 3.4.1. HLB Value

Through the calculation of the HLB value of the synthesized polymer, the hydrophilicity and hydrophobicity of the synthesized samples can be theoretically determined, as shown in Table 3.

It can be seen from Table 3 that, with the increase of the monomer unit content in the side chain, the HLB value of the PBA-g-PDMAEMA samples decreased, signifying the enhanced hydrophobicity/lipophilicity. When the monomer unit content of the side chain was high—for example, the molar ratio of the side chain monomer to the main chain monomer was higher than 15:1—the HLB value gradually tended to be stable.

It was interesting that the HLB values were completely inversely proportional to the contact angle values mentioned above, showing a very good consistency. The above results indicate that the novel polymers synthesized in this study have a significant hydrophobic tendency, especially when the content of the side chain monomer unit is high, which will help to achieve the advantage of hydrophobic performance in an alkaline environment by adjusting the molar ratio of the main chain monomer unit and the side chain monomer unit.

#### 3.4.2. Evaporation Rate

**Figure 6:**
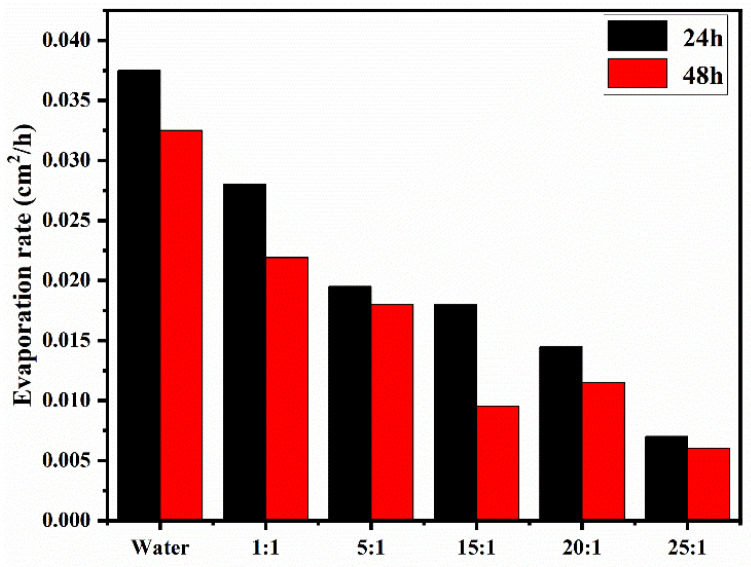
Evaporation rates of PBA-g-PDMAEMA aqueous solutions within 24 h and 48 h.

The enhanced hydrophobicity of the polymer in the aqueous solution helps to achieve water retention, inhibit water evaporation, and reduce drying shrinkage [45], which is of great significance for potential subsequent applications in cement-based materials. For cement mortar, the faster the evaporation rate of water in the capillary pores and the greater the additional pressure of the capillary pores, the more severe the volume shrinkage of the hardened cement paste, which will affect the later durability. Herein, the evaporation rates of pure water (for comparison) and five PBA-g-PDMAEMA aqueous solutions within 24 h and 48 h under the condition of pH = 13 are shown in Figure 6.

In Figure 6, it can clearly be seen that the evaporation rates of the polymer aqueous solutions were lower than those of the water sample within either 24 h or 48 h, indicating that the addition of the synthesized polymers can significantly inhibit the evaporation rate of water. In addition, with the increase of the monomer unit content in the side chain, the evaporation rates of polymer solutions gradually decreased. This trend shows a very good consistency with the results of the contact angle and the HLB value mentioned above, further signifying that the content of the responsive monomer in the side chain can significantly affect the evaporation rate of water in an aqueous solution.

All of the above results showed that the polymers synthesized in this study can respond under an alkaline condition, presenting the transition from hydrophilicity to hydrophobicity. Therefore, in an alkaline environment similar to cement mortar, these polymers could theoretically achieve excellent conversion from hydrophilicity to hydrophobicity and an internal hydrophobic effect of cement-based materials, which will be discussed in detail below.

### 3.5. Performance of Cement-Based Materials

#### 3.5.1. Water Absorption

**Figure 7:**
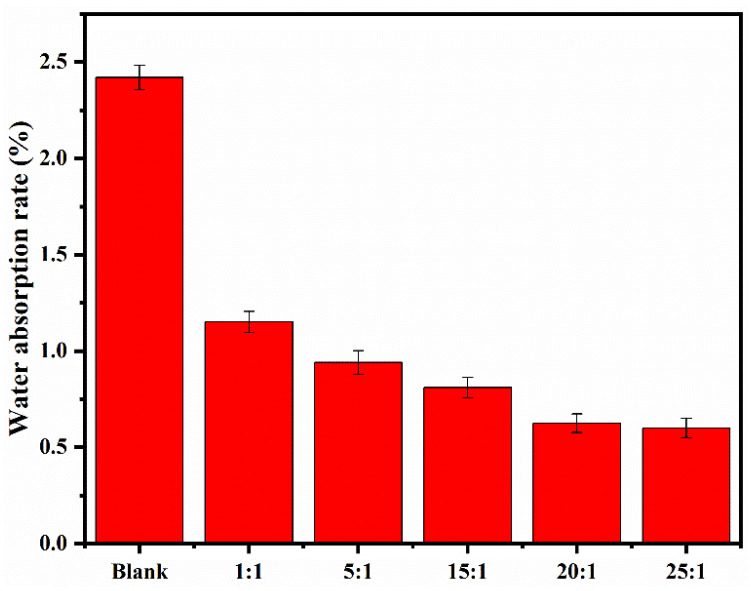
Water absorption rates of cement mortars with or without PBA-g-PDMAEMA.

To further probe the internal hydrophobic effect of the synthesized polymers on cement-based materials, the water absorption rates of cement mortars with or without five synthesized PBA-g-PDMAEMA samples were tested after a curing age of hydration for seven days, respectively, as shown in Figure 7.

As can be seen from Figure 7, the water absorption rates of cement mortars containing PBA-g-PDMAEMA were much lower than that of the blank sample, and in addition, with the increase of the monomer unit content in the side chain, the water absorption rates of cement mortars gradually decreased. When the molar ratio of the side chain monomer to the main chain monomer was 25:1, the water absorption rate of cement mortar containing synthesized PBA-g-PDMAEMA was the lowest, which was 75.2% lower than that of the blank sample. This indicates that under the alkaline environment of cement mortar, the synthesized PBA-g-PDMAEMA can change from hydrophilicity to significant hydrophobicity, which inhibits the entry of external water. Furthermore, it can be concluded that the water absorption rate of cement mortar containing PBA-g-PDMAEMA is closely related to the side chain length of PBA-g-PDMAEMA, implying that the PDMAEMA side chains in the comb-like PBA-g-PDMAEMA molecule is still the key to their function.

When PBA-g-PDMAEMA entered the interior of the cement mortar, due to the alkaline cement slurry system with a pH value of about 12.5, the tertiary amino groups in its side chain PDMAEMA were easy to lose protons, thus changing from hydrophilicity to hydrophobicity [46]. Additionally, its main chains were anchored on the surfaces of the cement particles by charge adsorption, which can reduce the surface energy and improve the dispersibility to help to stabilize the hydrophobic effect of PBA-g-PDMAEMA macromolecules. As a result, a large number of PBA-g-PDMAEMA macromolecules formed a hydrophobic film inside the cement mortar, which realized the hydrophobicity of cement-based materials from the inside to the outside, and thus greatly reduced the water absorption rate, which can resist external erosion to prolong the service life of cement-based materials [47,48].

#### 3.5.2. Dry Shrinkage

**Figure 8:**
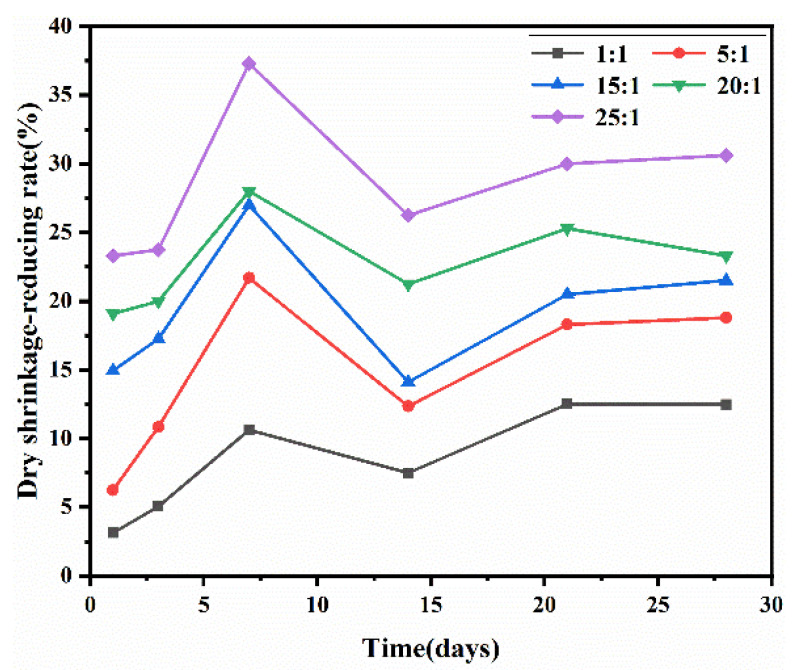
Dry shrinkage-reducing rates of cement mortars containing PBA-g-PDMAEMA.

The dry shrinkage of cement-based materials is closely related to the evaporation and migration of water, and herein, the dry shrinkage data of cement mortars containing the synthesized PBA-g-PDMAEMA samples at 1, 3, 7, 14, 21, and 28 days were tested, respectively. The dry shrinkage-reducing rates of each sample at different ages were calculated, as shown in Figure 8.

In Figure 8, the dry shrinkage-reducing rates of all samples showed a peak at seven days, possibly because the PBA-g-PDMAEMA macromolecules hindered the transformation of ettringite to monosulfide-hydrated calcium sulfoaluminate, which was prone to dry shrinkage, leading to more a significant shrinkage-reducing effect of cement mortars mixed with PBA-g-PDMAEMA in the early stage [49,50]. After seven days, the shrinkage-reducing rates decreased, as did the proportion of C–S–H, which was easy to shrink in the dry state as hydration products increased continuously with the hydration of cement. At 14–28 days, the shrinkage-reducing rates showed a stable trend.

Also from Figure 8, PBA-g-PDMAEMA samples showed a significant shrinkage-reducing effect on cement mortar, which was directly proportional to the side chain length of PBA-g-PDMAEMA. The longer the side chain length of the polymer sample, the higher the shrinkage-reducing rate of cement mortar. When the molar ratio of the side chain monomer to the main chain monomer was 25:1, the corresponding dry shrinkage-reducing rate of cement mortar exhibited the most significant shrinkage-reducing effect, reaching 37.3% at 7 days and 30.6% at 28 days. This indicates that the PBA-g-PDMAEMA synthesized in this study can significantly inhibit the drying shrinkage of cement mortar.

The above results showed that the application performances of PBA-g-PDMAEMA in cement-based materials were related to its comb-like molecular structure, which determined that the functional effect of PBA-g-PDMAEMA was played by the responsive behavior of its side chains. The longer the length of the side chain, the more helpful to the internal hydrophobic effect in cement-based materials, so as to achieve an excellent shrinkage-reducing effect and ultimately improve the durability of cement-based materials, which is of great significance.

#### 3.5.3. Mechanical Strength

**Figure 9:**
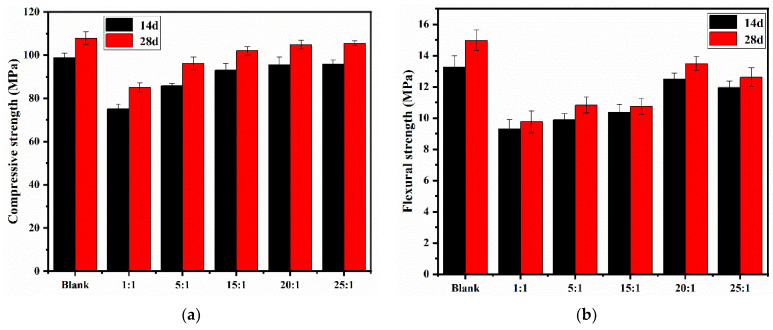
Mechanical strengths of cement mortars with or without PBA-g-PDMAEMA: (**a**) compressive strength; (**b**) flexural strength.

Figure 9 shows the compressive and flexural strengths of cement mortars with or without the synthesized PBA-g-PDMAEMA samples at 14 and 28 days, respectively.

From Figure 9, the strengths of cement mortars mixed with PBA-g-PDMAEMA were slightly lower than those of the blank sample, and the reduction of the flexural strength was slightly greater. Compared with the blank sample, the compressive strengths of sample 25:1 at 14 days and 28 days decreased by 3.1% and 2.1%, respectively, but the flexural strengths of the samples decreased slightly more. These results showed the same trend of the water absorption rate and the dry shrinkage rate of cement mortars, that is, the longer the side chain length of comb-like PBA-g-PDMAEMA was, the higher the mechanical strengths of cement mortars were.

It is speculative that the reason why PBA-g-PDMAEMA caused a slight decrease in the mechanical strengths of cement mortars may be that the addition of PBA-g-PDMAEMA reduced the additional pressure of the capillary wall and further increased the radius of the capillary, so that the porosity of the cement mortar increased, the pore structure became loose, and the compressive and flexural strengths both decreased [51,52]. In conclusion, the addition of PBA-g-PDMAEMA had a slight influence on the strengths of cement mortar, the compressive strengths of samples at 14 days decreased from 24.06% to 3.15%, while it decreased from 21.14% to 2.13% at 28 days. The flexural strengths of the samples at 14 days decreased from 30.02% to 5.89%, while it decreased from 34.83% to 10.02% at 28 days, and the influence on the compressive strength was only less than 5% when the side chain was longer (i.e., sample 25:1).

### 3.6. Hydration Behavior

**Figure 10:**
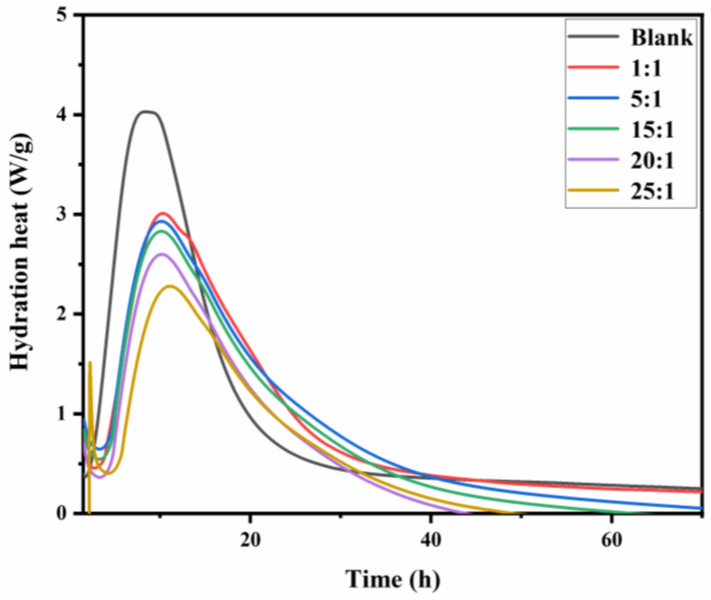
Hydration exothermic curves of cement pastes with or without PBA-g-PDMAEMA.

The process of the hydration and heat release of cement is closely related to the setting and hardening of cement paste, and herein, the hydration exothermic curves of cement pastes with or without the synthesized PBA-g-PDMAEMA samples are shown in Figure 10.

From Figure 10, the maximum exothermic peak of cement hydration for the blank sample appeared at about 8.4 h, while those for samples containing PBA-g-PDMAEMA all appeared at about 10 h, and even appeared at about 11 h when the molar ratio of the side chain monomer to the main chain monomer was 25:1. This indicates that, compared with the blank sample, the samples containing PBA-g-PDMAEMA showed a significant delay in the maximum hydration exothermic peak and a significant decrease in the hydration exothermic rate, which were attributed to the retardation of cement caused by the addition of the polymer [53]. During the first 20 h, sample 25:1 showed the lowest hydration peak height and cumulative heat release compared to the other four samples containing polymers. Moreover, after 40–60 h, in extreme cases, the hydration process of samples 15:1, 20:1, and 25:1 almost stopped, indicating that the addition of polymers significantly affected the cement hydration process. With the increase of the side chain length, the heat release rate gradually decreased, and the maximum hydration exothermic peak gradually appeared later. This is because the PBA-g-PDMAEMA macromolecules reduced the interfacial energy of the cement particles and the hydration products in the cement pore solution through their main chains’ adsorption on the cement capillary wall, and their side chains’ hydrophobic film wrapping on the surfaces of cement particles. Moreover, the longer the side chain length was, the larger the wrapping range was, and the stronger the internal hydrophobic effect was, which further hindered the hydration process. The wrapping of cement particles by PBA-g-PDMAEMA will increase the surface area and water demand of hydration products, resulting in insufficient free water for cement hydration [54].

### 3.7. Hydration Products

**Figure 11:**
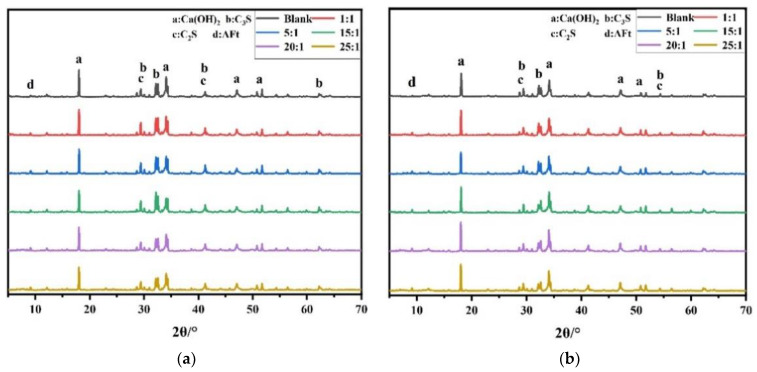
XRD patterns of cement pastes with or without PBA-g-PDMAEMA at: (**a**) three days; (**b**) seven days.

To further analyze the influence of PBA-g-PDMAEMA on the hydration products of cement-based materials, XRD measurements were performed, whereby cement pastes with or without the synthesized PBA-g-PDMAEMA samples hydrated at three days and seven days, and their XRD patterns are shown in Figure 11.

As can be seen from Figure 11, compared with the blank sample, the samples containing PBA-g-PDMAEMA showed almost the same diffraction peaks of the mineral phase, indicating that the addition of PBA-g-PDMAEMA did not change the types of cement hydration products. In Figure 11a, when the cement pastes hydrated for three days, the samples containing PBA-g-PDMAEMA showed significantly weakened diffraction peaks of CH compared with the blank sample, indicating that PBA-g-PDMAEMA inhibited the nucleation growth of CH at the early stage of cement hydration [55]. In Figure 11b, when the cement pastes hydrated for seven days, the samples containing PBA-g-PDMAEMA showed a slight enhancement in the diffraction peaks of AFt and CH. On the other hand, there was a slight weakening in the diffraction peaks of C_2_S and C_3_S compared with the blank sample, probably because the strong hydrophobic effect of PBA-g-PDMAEMA macromolecules in the later stage of cement hydration made more free water in the system, so that the unhydrated cement particles could be further dissolved and hydrated [56].

Overall, PBA-g-PDMAEMA had some effects on delaying the cement hydration, and hindering the early development of the microstructure of cement paste. The main reason is that the conversion of PBA-g-PDMAEMA macromolecules reduced the polarity of the cement pore solution, thus changing the concentration of some ions in the solution. As a result, the hydration process of C_3_S was delayed, and furthermore, the adsorption of PBA-g-PDMAEMA on the surfaces of cement particles inhibited the growth of CH crystals and hindered the formation of chemical bonds between hydration products [54].

## 4. Conclusions

A novel comb-like polymer PBA-g-PDMAEMA was designed and successfully synthesized by first the “main chain” (self-polymerization), then the “side chain” (graft copolymerization) method, whose molecular structure was proven to be the same as our design. Its main chain can hydrolyze under the alkaline environment of cement-based materials to produce anchoring groups that can adsorb on the surfaces of cement particles, and its side chains can achieve the conversion from hydrophilicity to hydrophobicity in an alkaline environment and produce an internal hydrophobic effect in cement-based materials.

The synthesized PBA-g-PDMAEMA showed a responsive behavior of changing from hydrophilicity to hydrophobicity when adding to the alkaline environment (pH value of 12–13) of cement-based materials due to good hydrophilicity in the pH range of 7–11, but good hydrophobicity in the pH range of 12–14. Moreover, the side chain length of this comb polymer directly affected its hydrophobicity. With the increase of the side chain length, the larger the contact angle, the lower the evaporation rate, the lower the HLB value, and the stronger the hydrophobic effect.

When the synthesized polymer was added to cement mortar, the water absorption rate of cement mortars decreased sharply and the dry shrinkage-reducing rate of cement mortars at each age increased sharply, and this trend became more pronounced as the side chain length increased. When the molar ratio of the side chain monomer to the main chain monomer was 25:1, the water absorption rate was the lowest, which was 75.2% lower than that of the blank sample, and the dry shrinkage-reducing rate at 7 days and 28 days can reach 37.3% and 30.6%, respectively. A thoroughly internal hydrophobic effect with almost no adverse effect on strength in cement-based materials was also achieved when the molar ratio of the side chain monomer to the main chain monomer was 25:1.

Another important finding of this study was that adding this polymer can effectively reduce the hydration exothermic rate and hydration heat and delay the maximum hydration exothermic peak, and this trend also became more pronounced as the side chain length increased. The produced internal hydrophobic effect helped to inhibit the formation of hydration products such as CH and the development of microstructures in the early stage, while there was no effect in the later stage. However, the potential weakness in this study was the strength of the cement mortar which mixed with the polymer. Only when the molecular weight was too high, the strength decline can be solved slightly. The way to make up for this disadvantage can be adding an early-strength agent or adding an external admixture, which can increase the early strength. In conclusion, the purposes of this study are to offer a new approach to prepare a novel comb polymer with a good internal hydrophobic effect on cement-based materials by self-driving under the cement alkaline environment, and to provide a promising alternative to hinder the erosion of external harmful substances, giving a useful insight into the mechanism of improving the durability and service life of cement-based materials.

## Figures and Tables

**Table 1:** Chemical and mineral compositions of reference cement/%.

Composition	SiO_2_	Al_2_O_3_	Fe_2_O_3_	CaO	MgO	SO_3_	Na_2_Oeq	f-CaO	C_3_S	C_2_S	C_3_A	C_4_AF
Reference cement	22.93	4.29	2.89	66.23	1.92	0.35	0.70	0.64	58.78	21.38	6.49	8.77

**Table 2:** Molecular weights of PBA-g-PDMAEMA.

Sample	PBA-g-PDMAEMA-1:1	PBA-g-PDMAEMA-5:1	PBA-g-PDMAEMA-15:1	PBA-g-PDMAEMA-20:1	PBA-g-PDMAEMA-25:1
Monomer ratio (DMAEMA:PBA)	1:1	5:1	15:1	20:1	25:1
Mw of copolymer (g/mol)	14,122	35,474	88,854	116,525	142,234
Mw of side chain (g/mol)	157	785	2355	3140	3925

**Table 3:** HLB values of PBA-g-PDMAEMA samples.

Sample	1:1	5:1	15:1	20:1	25:1
HLB value	11.0	6.5	4.8	4.5	4.4

## Data Availability

Data is contained within the article.
